# Understanding Inequalities in the Uptake of Supportive Care to Improve Practices in the Cancer Care Continuum

**DOI:** 10.3390/cancers14246053

**Published:** 2022-12-08

**Authors:** Jade Gourret Baumgart, Hélène Kane, Sylvie Pelletier, Karine André, Catherine Barbe, Thierry Lecomte, Yacine Sam, Nizar Messai, Emmanuel Rusch, Frédéric Denis

**Affiliations:** 1 EA 7505 Laboratory of Education, Ethics, Health, Faculty of Medicine, François-Rabelais University, 37 000 Tours, France; 2 Oncology Network Centre-Val de Loire (OncoCentre), 37 000 Tours, France; 3 League Against Cancer Departmental Committee of Indre-et-Loire, 37 000 Tours, France; 4 Regional University Hospital Centre of Tours (CHRU de Tours), 37 000 Tours, France; 5 Nutrition, Growth and Cancer, INSERM UMR 1069, University of Tours, 37 000 Tours, France; 6 EA 6300 Fundamental and Applied Computer Sciences, Polytechnic School of the University of Tours, 37 000 Tours, France

**Keywords:** cancer, supportive oncology care, health inequalities, health pathways, disease pathways, access to care, diagnosis announcement consultation, e-health

## Abstract

**Simple Summary:**

In view of its contribution to improving the quality of life of those affected by cancer and their survival rates, access to supportive oncology care is a major public health issue. Thus, inequalities in access to such care represent missed opportunities and impact the experience of the disease and the quality of life of those concerned. The aim of the present qualitative study was to gain insight into disparities in the uptake of supportive oncology care by users of oncology services. It revealed that significant variations in the uptake of such care are underpinned by identifiable disparities in their healthcare pathways. It provides some insights into the ways in which these inequalities in supportive oncology care uptake are constructed in complex ways, beyond informational and financial aspects.

**Abstract:**

(1) Background: While inequalities in the prevalence of cancer, access to care, and survival have been well documented, less research has focused on inequalities in the uptake of supportive oncology care. Given its contribution to improving the quality of life of people affected by cancer, access to such care is a major public health issue. The present study focuses on the access and uptake of those supportive oncology care services. (2) Methods: This study is based on qualitative research methodology, using a thematic analysis tree on NVivo© analysis software. First, an exploratory survey was conducted with users of oncology services, and professionals from these services and supportive oncology care. Then, individual interviews were conducted in June 2022 among people who are currently being treated or have been treated for cancer. (3) Results: The experiences of the 33 respondents revealed that significant variations in the uptake of supportive oncology care are underpinned by identifiable disparities in their healthcare pathways: in their assimilation of information, difficulties in accessing oncology care, personal reluctance and motivations, perceived needs and benefits, and use of other medicines. (4) Conclusion: This study aims to gain some insight into disparities in the uptake of supportive care in the Centre-Val de Loire region (France). Thus, it provides a better understanding of the complex ways in which these inequalities in supportive oncology care uptake are constructed.

## 1. Introduction

Inequalities in cancer prevalence and survival have been widely highlighted by various studies [1,2,3,4] that have shown social and territorial disparities [5,6]. Other studies have also highlighted inequalities in access to care, particularly with regard to delays in screening and initiation of cancer treatments (surgery, chemotherapy, radiotherapy, etc.) [7]. While these inequalities related to cancer care are well documented, little research has been devoted to inequalities in access to what are known as “supportive care” [8] and “rehabilitation” [9,10] in the cancer care continuum, which represents missed opportunities and also impacts the experience of the disease and quality of life of those concerned.

Supportive care is now defined as “all the care and support needed by patients, in addition to specific treatments, when available, throughout the course of serious illness” (AFSOS, 2022). In the context of oncology, “supportive oncology care” (SOC) aims to improve the quality of life of people affected by cancer, including patients and their families.

These SOC were officially introduced in France in the early 2000s via the 42nd Measure of the 1st Cancer Plan (2003–2007 Cancer Plan). The SOC offering covered by the French Social Security system was redefined in 2016 by INCa [11]. It currently includes pain management; dietary and nutritional monitoring; psychological support; social, family, and professional support; adapted physical activity; lifestyle advice; support for family members and caregivers; measures to preserve fertility; and management of sexuality disorders. Other treatments, such as social aesthetic care, are also included in this diversified range of services, although they are not currently included in this offering.

Beyond the positive impact of SOC on quality of life, which has been widely investigated [12,13,14], the use of SOC has been shown to improve survival rates among people facing cancer [15]. The second AFSOS Barometer [16], however, highlighted the fact that the term “supportive oncology care” itself is poorly identified by people affected by cancer, with only 34% of respondents being familiar with the term. However, another general population survey [16] showed that, when explained what SOC consists of, 76% of respondents considered this care to be as important as medical treatment for cancer.

Despite the emphasis placed by public policies on promoting SOC, notably via the 2021–2030 Ten-Year Cancer Control Strategy, the uptake of this care remains relatively low. Moreover, there are significant inequalities in uptake on a national level, which are reflected regionally. In the Centre-Val de Loire region, the SOC offer is mainly organised by hospital structures, the League Against Cancer Departmental Committees, and town and city boards. However, a survey coordinated by the Regional Cancer Network on SOC uptake [17] highlighted inequalities in information and access, determined by age, gender, and social background.

The SQVALD (*Projet pour l’organisation du parcours de Soins de support et la Qualité de Vie des patients avec une Affection de Longue Durée* (Project for organising the supportive oncology care pathways and the quality of life of patients with long-term conditions) project, funded by the Centre-Val de Loire region and which includes this study, aims to develop a new system for promoting SOC for people affected by cancer, based on the premise that people lack information about the services available to them. The aim of the project is to develop a web/mobile platform dedicated to the SOC offering in the Centre-Val de Loire region and to increase the rate of SOC uptake. In this context, this research, which is a preliminary study for the development of this new system, aims more specifically to gain insight into the inequalities in uptake of SOC in the Centre-Val de Loire region in order to, in turn, understand the extent to which—and the conditions under which—this system can help to reduce inequalities in access to this care. As such, it was necessary to more broadly analyse how SOC is perceived by users and how and why they integrate it into their care.

## 2. Materials and Methods

This work was conducted using qualitative research methodologies. First, exploratory research was conducted through participant observation and exploratory interviews. Then, to investigate the experiences of people directly affected by cancer in relation to SOC, individual interviews were conducted in June 2022 with users of oncology services in the Centre-Val de Loire region.

As part of the exploratory field study, the first two authors—Hélène Kane and Jade Gourret Baumgart—met with various professionals involved in SOC-related activities for exploratory interviews: oncology specialists, a nurse in charge of announcing cancer diagnoses, two professionals in charge of organizing SOC services in the Centre-Val de Loire region, a psychologist, a social worker, a dietician, a professional trained in adapted physical activity, a relaxation therapist, and three social aestheticians. In addition to these interviews, they attended social aesthetic treatments provided at the hospital and participated in adapted physical activity and sophrology sessions at League Against Cancer 37 premises. The purpose of the exploratory interviews and observations with the professionals was to understand their roles, depending on the profession, with regard to SOC and to explore in what ways and in what terms they talk about this care with people. A further dozen exploratory interviews were conducted with patients in the oncology department of the hospital. The aim of these informal interviews with patients was to collect initial results on inequalities in access and uptake of specific care in order to refine the interview guide for the formal interviews.

The interview guide was developed by the authors/researchers based on a narrative review of the French-language literature and the results of the exploratory survey. This ad hoc interview guide was structured around three lines of questioning: (1) personal experience with cancer, (2) individual experience of supportive oncology care, and (3) use of digital tools in individual healthcare pathways. The order of the questions was interchangeable to be able to build on the dynamics of each interview, and the questions were adapted to the contextual variations inherent to the participants’ individual journeys. Rather than starting from a theoretical definition of SOC, the approach consisted of understanding how this care is perceived, not only in relation to biomedical care but also in relation to leisure activities, well-being-related care, or practices related to other medicines.

The participants were recruited by the authors/researchers in a systematic way according to two methods: one was recruitment from within the day hospital services where participants were cared for and received their chemotherapy, and a second was recruitment in the premises of the Indre et Loire League Against Cancer Departmental Committee, where participants came to use SOC. Particular attention was paid to recruiting participants with a diversity of profiles in terms of gender, age, social background, and types of cancer.

The interviews were conducted by the first two authors; they conducted most of the interviews together and a few separately, face-to-face with the person affected by cancer. All interviews—with the exception of one by telephone—were conducted face-to-face, in accordance with the health regulations in force due to the ongoing COVID-19 pandemic. Interviews with oncology service users were continued until the data saturation threshold was reached [18].

The interviews were recorded and transcribed in full, and the transcripts were imported into the NVivo© analysis software (QSR International, Doncaster, Australia). This software was used to analyse the interviews using a thematic analysis tree [19] developed by the researchers in an iterative process through progressive adjustments between the research question and the interview data [20]. A few interviews were first coded in order to tweak the thematic analysis tree (Table A1), after which all the interviews were coded.

In accordance with the “Jardé” law (Decree no. 2016-1537 dated November 2016 and published on 17 November 2016, in the Official Journal of the French Republic), no regulatory approval was required for this study. After learning why they were included in the study, all participants were individually informed verbally, given an information sheet, and their consent was obtained. The survey was carried out so as not to disrupt departmental activities or cause difficulties for the users. Where it emerged from the discussions that interviewees had not been informed of the existence of SOC or had not made use of it, information concerning the SOC offering was provided at the end of the interview.

## 3. Results

In total, this study collected the experiences of 33 patients who were either currently being treated for cancer or had been treated for cancer at the University Hospital Center of Tours.

There were 19 female participants and 14 male participants, with an average age of 63.5 years, and they were affected by cancers of various types. These interviewees had diversified care paths, with not all of them having been informed of the existence of SOC; among those who were informed (Table 1); not all having used its services (Table 2); and whether or not they had mobilised other resources to cope with the situation (Table 3). 

### 3.1. Disparity in Access to and Uptake of Supportive Oncology Care Information

An initial observation was that most of the individuals affected by cancer interviewed were unfamiliar with the “supportive oncology care” terminology itself, even those who were aware of such services. Few participants claimed to be familiar with the term. Furthermore, the range of care encompassed by SOC was only partially identified. Those who best identified the content of this care offering were those who had actively sought out information about comprehensive cancer care options or who had been informed about them informally. Of the 33 participants, only 13 recalled being informed about SOC by someone on the oncology department team.

In principle, the users of oncology services are supposed to benefit from systematic information within the framework of the diagnosis announcement system. They are supposed to meet a few days after their cancer diagnosis with a diagnosis announcement nurse as part of a diagnosis announcement consultation. The aim of this procedure, which should take place at the beginning of the treatment process, is to inform people of their diagnosis, the treatment protocol, the possible side effects of biomedical treatments, and the SOC available. Some interviewees did not remember receiving this information from oncology professionals.

*“If you don’t ask, don’t look for it, nothing comes to you automatically, to tell you (...) There’s no tool.” [#19]*

Those who recalled being informed of SOC by oncology professionals generally had vague and incomplete memories, sometimes supplemented by having read the information sheet distributed to some. Several people said they were overwhelmed by the amount of information they received at the time.

*“I’ve not [received any information about SOC]. (...) Maybe in the early days, at the beginning of my treatment some nurses told me… [about SOC] (...) maybe, yes. But since then, perhaps I forgot (...) it was too much information.” [#26]*

Many also stated that when they received this information at the beginning of their treatment, they were shocked by the news and were more concerned about the seriousness of the disease and curative treatments. This was not a time when they were alert and receptive to the offer of supportive care. However, the information provided at the beginning of the course, while not always processed at the time, can sometimes be recalled later, particularly when patients hear about SOC again. Providing the information several times can thus facilitate the likelihood of SOC uptake. Moreover, several interviewees reported that the information that led them to SOC came from word-of-mouth, particularly from people with personal or professional experience relating to this care.

*“My son is a doctor, he discussed all this [supportive oncology care] with me and told me that if I needed it, I should take it.” [#11]*

This information, embodied in friendly or family relationships, attracts more attention from people. They represent an exchange of advice considered precious or comforting, of tips that help to forge a bond. One of the respondents [#20] mentioned how she herself regularly talked about SOC with other patients she met in the hospital, so that they too could benefit from it if they wished.

As information about SOC is not provided systematically and consistently, some people have doubts about their “right to receive it”. For example, one interviewee said she wondered whether this care was reserved for patients being followed for certain cancers only.

*“I came in for chemo, I saw people who were seeing psychologists, people who were seeing dieticians, and so on. I was never offered any of that (...) I was often with people who had breast cancer. I said to myself (...) “Well, in this department, are there distinctions made between cancers?” [#15]*

### 3.2. Disparity in Ease of Accessing Supportive Oncology Care

This survey highlighted how, in addition to being aware of the availability of SOC, various circumstances facilitate access to this care to a greater or lesser extent. One important constraint is the fact that the provision of SOC is concentrated in those cities in which hospitals have oncology departments. Those who live close to these cities find it easier to access them, while those who live far away generally rule out making a special visit, as they find the journeys they have to make for their curative care already burdensome and tiring. Certain healthcare situations are also varyingly conducive to accessing this care. For example, patients who have had a period of complete hospitalization are more likely to benefit from SOC offered in hospitals compared to those who have received care exclusively in day hospitals.

*“Every week here they have a kind of meeting, a staff meeting (...) everybody is represented, well the whole team: doctors, nurses, care assistants, art therapists, everybody participates. And the board discusses each patient’s case (...) and after that they ask: “Do you want to do this?”, or before they go to the meeting, they ask “Are there things you would like to do?”” [#16]*

Although SOC professionals working in the hospital explain that they work closely with the teams across departments and do outreach—going to rooms to offer care to patients—the likelihood of being offered care is reduced for those who come into the hospital less frequently. The teams are less familiar with their files and their potential needs. Furthermore, the various SOC professionals have limited time on the oncology wards, which reduces the likelihood that such patients will be able to benefit from this care directly before or after treatment without having to return specifically to the hospital. Moreover, returning to the hospital for SOC may involve transportation costs since medical transportation is not covered by the Social Security system if the only purpose of the trip is to receive SOC, which again illustrates the ‘optional’ nature of this care.

Moreover, fatigue from cancer and treatment discourages, or makes it impossible for some patients to participate in certain activities, including SOC. Paradoxically, although this care is intended to improve quality of life with the disease, particularly by minimizing the impact of treatment side effects, some patients perceive it as an extra effort they cannot afford given their state of fatigue. This intense physical fatigue can occur at random, leaving people fearful of failing and causing them to avoid activities that exert their physical abilities.

*“I have experienced this kind of drop in ability (...) for example, yesterday at the same time of day, I couldn’t have told you if I was going to come this morning [to the chemotherapy session] (...) I live from day to day.” [#31]*

Beyond this fatigue, people may have difficulty projecting the progression of their disease, especially when they suffer from cancers with a “poor prognosis”. The weight of these uncertainties can prevent them from drawing on resources like SOC. Some prefer to wait and see how their situation will progress and, in the meantime, do not feel they have the psychological availability necessary for them to invest in this care. This was true for one interviewee [#20], who expressed their desire to maintain a “normal life” for as long as possible.

*“In the immediate future, not so much, because I would like to try—not to forget, because we have this disease—but to get out of this obsession, if I can say that…” [#20]*

The fatigue associated with the disease and all that it implies means people can quickly become discouraged when the procedures become more complex, and they must multiply their efforts to access SOC. The difficulties in accessing this care echo other difficulties encountered over the course of cancer care and administrative procedures—and for which users can be helped by a social worker as part of the SOC—such as requests for recognition of a long-term illness or for adjustment of working conditions. Some patients say they feel exhausted just trying to obtain what they are entitled to receive.

### 3.3. Disparities in Reluctance and Motivation to Use Supportive Oncology Care

Beyond the practical difficulties, some people are reluctant to use SOC: they do not dare, do not feel legitimate enough to ask for it, or they feel their need is less than others’. These various cases illustrate how using supportive care requires the effort to at least “take the first step”. Some people do not feel able to seek this care on their own and wait to be reached out to, refusing to take the step of “asking for it”. This was true for one woman who enjoyed music but did not dare to take a music therapy session advertised on a poster, reasoning that she had not been “invited” [#15].

Reluctance may be linked to not wanting to return to a place that is associated with illness and the medical world, and sometimes even to situations that have been negative experiences.

*“First of all, I didn’t make the effort to get it [receive supportive oncology care at the hospital], because when I come here, I always want to leave quickly. (...) No one wants to be here.” [#17]*

Others emphasized their reservations about being with other people with cancer. They seemed to resent the fact that their identity was reduced to their illness and feared that being “among sick people” could have a negative impact on their morale.

*“You can’t know who you’re going to meet, and what state that person is going to be in compared to you (...) and during the conversation you find out that they won’t make it (...) you imagine that you have the same pathology as them. (...) So, you might have been in a good mood when you arrived, and by the time you leave in the afternoon you feel a bit down.” [#28]*

The experiences reported by several interviewees show that the support of family and friends can be decisive in overcoming some of these initial reservations. This was true for one interviewee, who was actively supported by her mother, who sought out information about SOC for her and encouraged her to use it (#27). This is especially true as, due to the high prevalence of cancer in the population, a significant number of respondents have people around them who have experienced cancer themselves and can thus derive some support from them in coping with the disease.

*“So, my aunt who had breast cancer gave me a lot of advice (...). She has good advice, because she’s been through it (...) Everyone is very worried, and she is the positive force who says: “Don’t worry, just... it will be okay.” [#21]*

However, some interviewees indicated that relatives were not always the best source of support, sometimes making awkward comments and gestures, not always being understanding, or sometimes being an additional burden. Indeed, sometimes interviewees said they had to deal with their relatives’ pain and worries in addition to their own difficulties. Taking care of loved ones prevented some patients from focusing on their own well-being.

*“With everything that has happened in the last six months, I spent more time reassuring my husband, for example, and I realized after a while that, well, we each have our cross to bear.” [#13]*

One interviewee explained how, in addition to family and friends, they found support within their religious community, in which they were already involved before the onset of their illness [#26]. Others explained how they had joined exchange groups, either discussion groups run by dedicated associations or groups on social networks. Another explained how she had joined a Facebook group in which she found support—peer support—in the form of advice and encouragement specific to her cancer.

*“In fact, I find myself in a group where we all have the same thing (...) I like being in this group (...) because I learn a lot of things, it’s them who have explained a lot of things to me too (...) And we are very close-knit, it’s like being sisters (...) as soon as one of us isn’t feeling good, she tells the group and fifty or so others will respond.” [#2]*

### 3.4. Disparity in Individuals’ Perceived Needs and Benefits of Supportive Oncology Care

Another source of disparity lies in the perceived need to find new resources and the perceived benefit of turning to SOC. Evaluating these needs is a particularly subjective process, especially since such care is perceived as optional, sometimes as a luxury. While some patients identify multiple needs engendered by the disease, others claim to find the comfort they need in their daily activities and with their loved ones, or even in their “quiet little life”.

*“Yes, I walk less. And my husband can’t come with me as he has problems with his feet. But we have a big garden, so I’m not cooped up (...) I like the calm. And I keep busy (...) with my house (...) cooking and making meals.” [#25]*

This can also apply to those who live in a house with a garden where they can do gardening or DIY, those who are used to going away for the weekend, especially if they have a second home, or those who have a pet.

A lack of interest in SOC may also be related to a lack of knowledge about it, preventing some patients from identifying how SOC might be appropriate for their situation and how it might enhance their well-being.

*“They didn’t suggest that I see a dietician, and considering my [thin] build, I don’t think I need to go on a diet, quite the opposite! My focus is rather on maintaining my weight” [#20]*

Others feel the need to find resources in this distressing context but do not consider that the SOC proposed can provide what they need. The question of identifying a need for psychological support in the face of cancer and the benefit of seeing a psychologist is raised in very inconsistent ways.

*“I was asked if I wanted to see a psychologist. What am I going to do, go and talk alone to someone who has nothing to do with my life? That’s not really my thing.” [#5]*

Many of the interviewees admitted to having found their cancer ordeal challenging, finding it difficult to accept, and having had to face dilemmas in managing it, but nevertheless did not feel that they needed psychological support.

*“I don’t need one [a psychologist]. I feel good mentally. Well, I have the odd moment… [sighs] when its more difficult than others (...). But you see, I don’t have the impression that I have a terminal illness (...). I hope that what I’ve been doing for the past few months will pay off, and that I’ll get through it.” [#14]*

Negative representations are specifically associated with consulting psychologists, which is seen by some as an admission of weakness and an inability to cope with the disease. Consulting a psychologist amounts to admitting that the cure for cancer is uncertain or even shows a lack of confidence in the skills of oncologists.

### 3.5. Disparities in the Use of Other Medicines

It also emerged that many of the respondents—whether they used SOC or not—used other medicines in their healthcare: osteopathy, acupuncture, foot reflexology, reiki, shiatsu, micro-physiotherapy, myotherapy, hypnosis, fasting, naturopathy, homeopathy, energy therapists, magnetizers, burn healers, etc. Some were probably confident of the benefits of these practices, which they used in addition to their allopathic medical treatments to deal with the cancer. These practices may have been used before the onset of their cancer, sometimes for a long time, as in the case of one interviewee who said she had been treated in this way since childhood [#9]. The others generally stated that they had learned about the various alternative practices, as with SOC, by word of mouth or after having done a lot of research on what could be beneficial to them. This was true, for example, for one interviewee who adopted a specific diet after reading a book on the subject [#13].

Several people used a combination of treatments in addition to their allopathic medicine, using both SOC and other medical practices. Beyond the quest for well-being, these individuals seemed to want to “give themselves every chance”, sometimes to the point of continuing sessions despite the financial commitment, organizational constraints, and discomfort or even pain they may engender. This was true for one interviewee, who hoped acupuncture would benefit her health, and having realized that the needles were painful for her skin, which had been made very sensitive by the chemotherapy treatments, indicated that she had nevertheless continued acupuncture for chemotherapy [#2]. Using as many resources as possible, such as alternative medicine, or SOC, can be considered a way of taking charge of one’s health.

*“I don’t know if this is much of a contribution, but the results showing the progression of my illness are still very positive, so I say that at the very least, this is my way of participating.” [#13]*

This being the case, other respondents explained how they turned to other medicines without really “believing in them”, but rather because they brought them comfort, in particular “the fact of talking to people” [#15], who gave them time and space to express themselves, which they could not always get from professionals in oncology departments or from those around them.

Such use of other therapies is not always undertaken in consultation with oncologists and other health professionals. However, one respondent explained that she had discussed it with her oncologist beforehand—and then with her pharmacist—asking them, “Is homeopathy good? Naturopathy?” [#2]. Similarly, one interviewee said that he had asked his doctors for permission to use other products and services that he himself described as “comfort products” [#19]. However, some people keep it a “secret” or do not mention it to their oncologist. For example, one interviewee who was seeing an acupuncturist and a magnetizer admitted that she had never talked about it with her oncologist and “hadn’t really thought about doing so” [#18]. Some probably avoid divulging such information because they have internalized the idea that these medicines are not accepted by everyone and thus fear the doctor’s reaction. As such, interviewees who said they did not use alternative medicines suggested that their use thereof may seem to reflect a lack of confidence in the care provided by allopathic medicine, or even reduce their chances of recovery.

*“I relied exclusively on the hospital, on what the hospital offered me.” [#4]*

This study revealed significant heterogeneities in the uptake of SOC, underpinned by identifiable disparities in the respondents’ healthcare pathways, concerning access to and assimilation of information about SOC, difficulties in accessing this care, reluctance and motivation to use it, perceived needs and benefits, and use of other medicines.

## 4. Discussion

In addition to the figures documenting inequalities in the uptake of SOC in the Centre-Val de Loire region, this investigation of people affected by cancer provides insights into the non-uptake of this care and the ways in which these inequalities are formed.

### 4.1. Diverse Relationships with the Disease

One aspect highlighted by this survey is that access to SOC is based on the different attitudes people adopt in relation to their cancer. The onset of a chronic disease such as cancer represents a biographical rupture [21], forcing people to reconstruct their identities and their daily lives. This biographical rupture takes on particular forms in the case of cancer, which is frequently associated with negative cultural representations: those of an insidious, degrading, and painful disease [22,23]. These representations position cancer as the “enemy to be fought” exhorting those affected to “fight against the disease” to be “strong” to “fight” and to “keep their spirits up” [24]. This fighting rhetoric, which was used in different ways by the interviewees, generates various attitudes toward SOC. This can make the uptake of psychological support difficult to accept, since it can be perceived as an admission of weakness. In contrast, engaging in adapted physical activities or following social aesthetic advice can be more easily integrated within the idea of “arming oneself against cancer”.

To cope with their cancer, the interviewees have taken positions other than the fighting stance [25]. Some people, especially the elderly, seek instead to accept the limitations and uncertainties associated with the disease in order to build a peaceful “living with” approach. This attitude favours the use of SOC if they are recognized as pleasant and comforting, such as sophrology and reflexology, for example, but also tends to lead to a renunciation of any efforts to engage in new activities. Some patients express forms of withdrawal into their immediate environment, into familiar places and activities that are socially differentiated, involving lots of trips and social relations, or, in contrast, confined to daily domestic activities.

Recovering from the biographical disruption of cancer can mean, for some, striving to “be like you were before” by minimizing the impact of the disease, while others seek to gain some benefit from the experience as a way of self-fulfillment [26]. This positive reinvestment of a distressing experience leads them to accept the presence of other sufferers more easily, who are perceived as having an experience and understanding to share [25]. The question of the body’s relationship to the disease is also central to this relationship. Social aesthetics and adapted physical activity can have a positive impact on the relationship that people affected by cancer have with their bodies. However, the reshaping of one’s body image during the disease and feelings of failure or degradation are sometimes such that they inhibit participation in certain activities, including those proposed by SOC.

The attitudes people hold towards their cancers, guided by gender and age patterns [27], also relate to how they shape their roles as patients. They can “trust” and accept what is offered to them, but they can also seek to “be actors in their program”, which can result in more demands on SOC or even in a cumulative use of different resources. It is also important to note that there are constraints on people’s ability to assume their role as patients: some have to look after relatives, and the disparities between the people around them can increase inequalities in the face of cancer [28,29].

### 4.2. Heterogeneity of Relationships to Medicine and the Healthcare System

The results also show that the likelihood to use SOC is determined by differentiated relationships with the medical world [30]. Some patients essentially expect curative, technical, and efficient care and do not consider that “comfort” or “well-being”-related care is a matter of medical responsibility. Preferring that professionals focus on curative care, they may either consider such supportive care to be of little use or prefer to seek other sources of care or comfort. For others, in contrast, SOC contributes to the quality of their cancer care, and using SOC gives them better care. This differentiation in the perceived role of SOC is maintained by the ambivalence of the healthcare system towards this care, which is not delivered systematically but instead proposed according to the individual’s means, situations, and needs, as identified by professionals. It is essential to note that non-use of the SOC proposed by the hospital encompasses a variety of situations, with some people foregoing it while others simply choose to use comparable resources outside of the hospital setting.

Reluctance to turn to SOC is particularly associated with negative experiences in hospitals in the presence of healthcare professionals. Interviewees’ accounts of receiving their diagnosis (evoking the word ‘cancer’ in particular) and of being informed of the medical uncertainty of their prognosis and even of the treatments themselves illustrate the fact that the ‘ordinary violence’ that is both inherent and intrinsic to cancer care [31] is sometimes experienced by people as being particularly intense: traumatic even. Faced with these negative experiences, positions are mixed, since some will prefer to avoid supportive care because it is presented to them by these same professionals in these same services, thus reminding them of unpleasant moments, while others will find a form of compensation and comfort in this fact.

Differences in the ways people relate to the healthcare system are also illustrated in their relations with healthcare professionals, some of whom question professionals, or even deftly negotiate care, and obtain more information [4,32], while others do not feel entitled to interfere in the care they receive. The differences in these therapeutic relationships mean that some people seek supportive care while others do not allow themselves to “claim” it. Accessing SOC services, such as psychological support, social aesthetics, or hypnosis, also implies recognizing vulnerabilities and weaknesses and accepting a care relationship. Gender constructs favour women who, in dealing with illness, tend to focus on “taking care of themselves”, while men, by minimizing their symptoms, tend to undermine their ability to implement care plans that are as closely as possible aligned to their needs [27].

Our results also emphasize the fact that speaking about access to SOC without considering the other resources people use (personal activities, other therapies, family, and friends, etc.) portrays a reductionist representation of certain disease trajectories. SOC services raise questions regarding the boundaries between different types of medicine. These are part of the ambivalence of the medical world—where knowledge and therapeutic practices belong to the field of biomedicine—concerning certain practices pertaining to non-conventional medicine [33,34]. The French Medical Board itself defines four types of alternative and complementary medicine [35]. This work has demonstrated the fact that medical and hospital settings are increasingly using people to provide care that falls under the scope of other medicines [36]. For example, patients undergoing radiation therapy are likely to be recommended “off the record” by some professionals in the oncology departments to see a burn healer or magnetizer to relieve the pain of radiation burns [34]. In this respect, SOC can be considered a gateway to alternative medicine in health services. The SOC offer proposed by oncology services and League against Cancer Departmental Committees includes resources derived from other therapies such as sophrology, music therapy, and art therapy, therefore giving these a certain form of legitimacy [37]. The increased use of cancer care does not necessarily imply access to new resources, but access to alternative therapeutic resources in a supervised context. It also represents a transition to a new model of care that could be applied to other pathologies beyond oncology [38].

### 4.3. Limitations

At the time of the interviews, the people interviewed were in very heterogeneous situations in terms of their care pathways: at the beginning of treatment after a recent diagnosis or having undergone treatment for several months or years, with a more or less positive, or even poor, cancer prognosis, with or without metastases, suffering various side effects of treatment, etc. These disparate situations influenced the quality of the care provided and undeniably influenced the interviewees’ comments. As a result, this work offers reflective feedback on the use of SOC in the specificity of people’s disease trajectories, which must be situated in these specific contexts [20]. However, this research has thus enabled a diversity of situations to be explored, highlighting a set of disparities that explain the inequalities in SOC uptake.

Another limitation of this work relates to the subject of “supportive oncology care” itself, which encompasses a disparate set of care and activities that our interviewees perceived as being separate. As this category did not always make sense to our participants, it is not appropriate to talk about this type of care in a general way. In particular, questions about “measures to preserve fertility” and “managing sexual dysfunction”—which are included in the SOC offering—were not discussed with interviewees. The context of the interviews, which took place in hospital rooms and were sometimes disrupted by outside interruptions related to patient care, was not appropriate for exploring these issues, and none of the interviewees addressed them spontaneously. However, questions relating to access to information on SOC, dietary and nutritional follow-up, psychological support, social, family, and professional support, adapted physical activity, and advice on healthy living were systematically explored.

The issue of cost or free access to SOC was little mentioned by the interviewees, who instead raised other practical difficulties and reluctance. In the wake of a body of research on the accessibility of care [39], our work contributes to showing how inequalities in access to SOC are constructed in a complex and discontinuous manner, beyond the sole questions of information and financial coverage by the Social Security system. Information and free access are not enough to generate equal access, and our work duly shows that uptake of SOC particularly depends on subjective constructions of relationships to illness, to the health system, and to medicines.

The integration of alternative care provision through SOC is likely to reinforce inequalities in access to care for people with cancer, and attention must be paid to enabling equitable access to the different types of SOC.

## 5. Conclusions

This work shows that inequalities in the uptake of SOC are complex, not only regarding disparities in terms of information and difficulties in accessing this care but also in relation to illness and medicine, which are interconnected in a unique way. It seems difficult to act on certain factors, such as geographical distance or the heterogeneity of patients’ social environments. On the other hand, it is necessary to think about and implement—among people affected by cancer—new devices, including web/mobile devices, to support the existing diagnosis announcement procedure. This would ensure effective, systematic, and adequate promotion of SOC among all users of oncology services. In this way, mobilizing digital tools to develop web/mobile platforms for the use of those concerned, such as those that present accessible SOC services, is an opportune way to provide information [40]. It is nevertheless important, in order to maximise the chances of people owning the device, to ensure that interfaces are developed ergonomically and inclusively, particularly in terms of the representations they convey of people affected by cancer (people with visible side effects of biomedical treatments, etc.), and to take into account the fact that people have different levels of health literacy. To best meet the challenge of social acceptability of the device, it is preferable to avoid referencing the hospital and medical world, which are often associated with negative aspects of the disease experience. Finally, it is important to consider the integration of these new devices into the healthcare pathway of people affected by cancer and to implement these new devices for promoting SOC in addition to—and not in place of—verbal and printed communications.

## Figures and Tables

**Table 1:** Information on how interviewees with cancer received or did not receive information about SOC.

	Recalls Being Informed about Supportive Oncology Care	
Interviewee’s Information	by Oncology Services Teams	by Other Means
No. 1, female, 75 years, cancer	no	
No. 2, female, 50 years, breast cancer diagnosed in early 2022	yes	
No. 3, male, 73 years, cancer of the digestive system diagnosed by the end 2021	no	
No. 4, male, 85 years, ENT cancer diagnosed by end 2021	no	
No. 5, female, 68 years, stomach cancer diagnosed in 2019	yes	
No. 6, female, 64 years, cancer in early 2022	yes	
No. 7, male, 70 years, cancer	no	
No. 8, female, 67 years, metastatic ENT cancer diagnosed in 2018	no	
**No. 9, female, 54 years, colon cancer diagnosed by end of 2021**	**no**	**Yes, a co-worker who had had breast cancer herself told her about what was offered at the League Against Cancer**
**No. 10, female, 45 years, cancer**	**no**	**yes**
No. 11, female, 75 years, stomach cancer	no	Yes, her son is a doctor and told her about it
No. 12, female, 73 years, biliary tract cancer diagnosed in 2020	yes	
No. 13, female, 65 years, myeloma	Yes, remembers being told about it during the presentation of the treatment protocol	
No. 14, female, 74 years, history of meningioma diagnosed in 2010 and breast cancer	Yes, remembers a nurse giving her a folder with this information in it	
No. 15, female, 75 years, colon cancer diagnosed in 2021 with liver metastases	no	Yes, she saw that supportive oncology care was being offered to other users
No. 16, male, 51 years, leukaemia	yes	
No. 17, female, 50 years, breast cancer diagnosed in 2020	Yes, in a diagnosis announcement consultation	
No. 18, female, 49 years, brain tumour by the end of 2020	no	
**No. 19, male, 55 years, cancer, diagnosed in early 2020**	**no**	**Yes, by his wife who, was an oncology nurse, told him about it**
No. 20, male, 75 years, myeloma diagnosed in 2010 and start of treatment in 2022	no	
No. 21, female, 43 years, breast cancer	-	Yes, because of her professional activity as a medical secretary
No. 22, female, 66 years, history of breast cancer diagnosed in 2007 and bone metastases in the femur diagnosed in 2016	yes	Yes, her sister told her about what was offered in a hospital in Paris
No. 23, male, 47 years, pancreatic cancer diagnosed in 2019	no	yes
no. 24, female, 54 years, pancreatic cancer with ovarian metastasis diagnosed in 2020	yes	
No. 25, female, 81 years, cancer, diagnosed in 2019	no	
No. 26, male, 65 years, rectal cancer diagnosed in 2018	no	
**No. 27, female, 43 years, breast cancer diagnosed in 2020**	**yes**	
No. 28, male, 66 years, liver cancer diagnosed in 2021	no	
No. 29, male, 67 years, pancreatic cancer	no	
No. 30, male, 78 years, oesophageal cancer	no	
No. 31, male, 78 years, history of prostate and renal cancer diagnosed in 2007 and pancreatic cancer diagnosed in 2022	-	Yes, because of his former professional activity as a doctor
No. 32, male, 68 years, metastatic colon cancer diagnosed in 2020	Yes, remembers being given a folder with this information	
No. 33, male, 48 years, colon cancer diagnosed in early 2022	yes	

Information related to interviewees recruited via the Indre et Loire League Against Cancer Departmental Committee is presented in bold, while information about interviewees recruited through day hospital services is presented in standard font. Of the 33 participants interviewed, 22 in total recalled being informed about SOC; 13 recalled being informed about SOC by a member of the oncology service team, while nine recalled having accessed the information by other means.

**Table 2:** Information on how interviewees with cancer accessed SOC.

User No.	Reported Having Accessed Supportive Oncology Care					
	Dietary and Nutritional Monitoring	Psychological Support	Social, Family, and Professional Support	Assistance in the Practice of an Adapted Physical Activity	Well-Being Care	Other
No. 1						
No. 2	Dietician at the hospital					
No. 3						
No. 4	Dietician at the hospital					
No. 5					Social aesthetician at the hospital	Hair prosthetist
No. 6			Social assistant at the hospital			Hair prosthetist
No. 7						
No. 8	Dietician at the hospital					
**No. 9**	**Dietician at the LAC37**	**Psychologist at the hospital**	**Social assistant via her health insurance**	**Adapted physical activity sessions at the LAC37**	**Social aesthetician, image advice workshops, sophrology, and music therapy at the LAC37**	
**No. 10**	**Dietician at the LAC37**	**Psychologist at the LAC37**	**Social assistant at the LAC37**		**Social aesthetician and sophrology at the LAC37**	
No. 11	Dietician at the hospital					
No. 12						Hair prosthetist
No. 13	Dietician at the hospital					
No. 14					Social aesthetician at the hospital	Hair prosthetist
No. 15						
No. 16			Social assistant at the hospital		Social aesthetician and hypnosis sessions at the hospital	
No. 17					Social aesthetician at the hospital	Hair prosthetist
No. 18			Social assistant at the hospital			
**No. 19**	**Dietician at the LAC37**			**Adapted physical activity at the LAC37**	**Sophrology at the LAC37**	
No. 20						
No. 21					Social aesthetician at the LAC37	
No. 22				Participated in sessions at the LAC37	Social aesthetician and sophrology sessions at the LAC 37	
No. 23	Dietician at the hospital	Psychologist at the hospital	Social assistant at the hospital		Sophrology and Chi Cong at the LAC 37	
No. 24	Dietician at the hospital					
No. 25						
No. 26						
**No. 27**	**Dietician at IETO 37**	**Psychologist at the LAC37**		**Adapted physical activity at the LAC37**	**Socio aesthetician, image-advice, and COGITE workshops at the LAC37**	
No. 28						
No. 29	Dietician at the hospital					
No. 30	Dietician at the hospital					
No. 31						
No. 32	Dietician at the hospital					
No. 33	Dietician at the hospital		Social assistant at the hospital		Social aesthetician at the hospital	

Information related to interviewees recruited via the Indre et Loire League Against Cancer Departmental Committee is presented in bold, while information about interviewees recruited through day hospital services is presented in standard font. Of the 33 participants interviewed, 10 reported having accessed none of the supportive care, 11 reported having accessed one type of supportive care, and 12 reported having accessed more than one type of supportive care; the maximum number of reported types of supportive care accessed was five of the six. The supportive care type declared most frequently accessed was dietary and nutritional monitoring, while psychological support was declared to be less frequently accessed among the participants interviewed.

**Table 3:** Information on which other activities, resources, and medicines interviewees mobilised.

User No.	Reported Having Had Recourse to Other Activities or Resources to Cope with the Situation	Reported Having Used Other Medicines to Cope with the Situation
No. 1		
No. 2	Joined the private Facebook group “Les triplettes” which is for women with triple negative breast cancer	Homeopathy, acupuncture, and foot reflexology
No. 3	Walks, bikes, and is a gym member	Recently used a burn healer for shingles
No. 4	Walks and gardening	
No. 5	Gardening, reads books, and plays games on her tablet	
No. 6	Cooking and genealogy research	
No. 7		
No. 8	Gardening and playing computer games	
**No. 9**	**Walks and gardening**	**Magnetizer**
**No. 10**	**Cooking, baking, gardening, and genealogy research**	**Kinesiology**
No. 11	Walks the dog, gardening, and reads books	
No. 12	Plays games on her tablet	
No. 13		
No. 14	Reads books	
No. 15	Walks, pilates, and listens to music	Acupuncture and reiki
No. 16	Walks, photography, and stationary exercise bike	
No. 17		
No. 18		Acupuncture and magnetizer
**No. 19**	**Walks about 5 km a day, DIY, and mechanical work**	
No. 20	Belongs to a walking group and a choir, gardening, and reads books	
No. 21	Joined a private Facebook group for people with breast cancer and walks 30 min/day	
No. 22	Watches medical series on TV	Myotherapy
No. 23	Plays the piano, listens to music, and gardening	
No. 24		Previously used acupuncture and seen an energy therapist, and continues to use a magnetizer and naturopathy
No. 25	Cooking, television viewing, and scrabble	
No. 26	His faith kept him actively involved in his religious community	
**No. 27**		**Energy therapist, acupuncture, micro-kinesis, and her spouse healed her burns**
No. 28	Walks and watches television	
No. 29	Gardening	Burn healer
No. 30	Watches television	
No. 31	Reads books, watches television, goes away on weekend breaks, and consulted a psychologist	
No. 32	Reads books, goes for walks, swims in his pool, crafts and mechanics, and rides his motorcycle	
No. 33	Swims in his pool and DIY	

Information related to interviewees recruited via the Indre et Loire League Against Cancer Departmental Committee is presented in bold, while information about interviewees recruited through day hospital services is presented in standard font. Of the 33 participants interviewed, 26 reported having had recourse to other activities or resources to cope with the situation, and 10 reported having used other medicines to do so; seven reported having used activities, resources, and other medicines, while five reported they had recourse to none.

## Data Availability

The data are fully available and will be shared upon request with the co-first authors.

## Appendix A

**Table A1:** Thematic analysis tree exported from NVivo© analysis software.

Items	Number of Interview Transcripts Coded for This Item
Use of non-SOC resources	
leisure activities	25
family circle	24
circle of friends	16
religious community	1
network of associations	6
leather people affected by the cancer	11
other medicines	10
Use of SOC	
informed by oncology service teams	13
informed by other means	9
difficulties accessing them	15
reluctance to use	13
perceived interest	12
Experience of the cancer journey	
attitude towards the disease	29
uncertainty regarding the evolution of one’s health status	14
side effects preventing participation in activities	19
relationship with one’s body	9
negative experiences	13
positive experiences	16
